# The Role of Macrophage-Derived Netrin-1 in Inflammatory Diseases

**DOI:** 10.3390/biom15070921

**Published:** 2025-06-23

**Authors:** Yi Wu, Zhiying Liu, Peiqi Xu, Kai Yin, Shengjun Wang

**Affiliations:** 1 Department of Laboratory Medicine, Jiangsu Province Engineering Research Center for Precise Diagnosis and Treatment of Inflammatory Diseases, The Affiliated Hospital of Jiangsu University, Zhenjiang 212001, China; wy1278694715@163.com (Y.W.);; 2 Department of Immunology, Jiangsu Key Laboratory of Laboratory Medicine, School of Medicine, Jiangsu University, Zhenjiang 212013, China; 3 Department of General Surgery, Affiliated Hospital of Jiangsu University, Institute of Digestive Diseases, Jiangsu University, Zhenjiang 212001, China

**Keywords:** macrophage, netrin-1, inflammatory diseases, immune regulation

## Abstract

Macrophages are multifunctional immune cells distributed throughout the whole body, and they have functions in antigen presentation, phagocytosis, killing, and immune regulation. As the most widely studied molecule in the netrin family, netrin-1 plays a key role in neuronal navigation, angiogenesis, and cell survival. Macrophage-derived netrin-1 not only regulates neurovascular regeneration through ligand–receptor binding but also influences macrophage phenotypes by modulating polarization, thereby achieving the purpose of promoting or repairing disease damage. In this review, we will summarize the recent research advances on the role of macrophage-derived netrin-1 and its receptors in a variety of inflammatory diseases and cancers.

## 1. Introduction

Netrin-1 is a laminin-like related secreted protein that was originally described as a guiding signal in the developing central nervous system [1]. Netrin-1 is expressed in various tissues, including the brain, lung, heart, liver, intestine, and kidney [2]. It is mainly expressed by nerve cells [3], tumor cells [4,5], macrophages [6], and endothelial cells [7]. In recent years, studies indicate that netrin-1 participates in angiogenesis, organ formation, inflammation regulation, and cancer progression by binding to its receptors [8]. Macrophages are involved in innate and adaptive immunity and are key regulators of normal homeostasis and pathology. However, little is known about the effect of macrophage-derived netrin-1 on macrophage functions or the local microenvironment in inflammatory diseases [9]. In this review, we will discuss the self-regulation and regulation of netrin-1 in the disease microenvironment from three perspectives: acute inflammatory diseases (ischemic stroke, acute lung injury, and acute kidney injury), chronic inflammatory diseases (diabetes, atherosclerosis, abdominal aortic aneurysm, osteoarthritis, pulmonary fibrosis, and endometritis), and cancer (glioblastoma and non-small cell lung cancer). We will also explore the regulatory role of pericytes (Table 1), which could contribute to the development of new treatments for diseases.

## 2. Origin, Polarization, and Function of Macrophages

**Figure 1:**
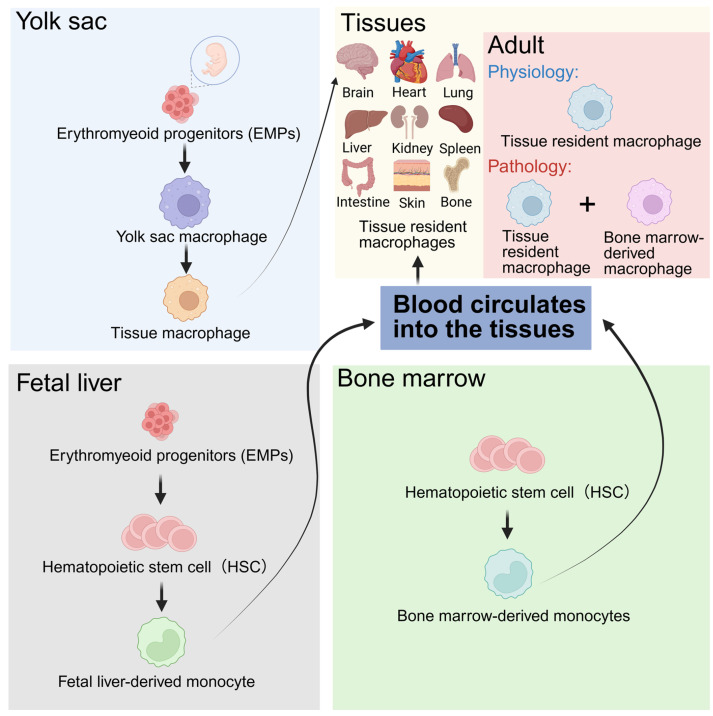
The origins of macrophages. Macrophages in developing tissues are mainly derived from the yolk sac and fetal liver. After maturation, macrophages colonize and maintain self-renewal in various tissues. In adulthood, macrophages are generated from monocytes in the bone marrow and migrate to tissues through the blood circulation.

The origin, migration, and development of macrophages is a complex process that shows a high degree of similarity between humans and mice [10]. During embryonic development, the erythroid–myeloid progenitor cells (EMPs) of the yolk sac differentiate in situ into macrophages, and this group of macrophages migrates to the brain tissue to become microglia [11,12]. The EMPs in the yolk sac then migrate to the fetal liver, where they differentiate into hematopoietic stem cells and later differentiate into monocytes, the precursors of macrophages, which are spread through the blood and colonize in the tissues [13]. Hematopoietic stem cells in the bone marrow can also differentiate into monocytes and enter the tissue to differentiate into macrophages. After adulthood, macrophages mainly originate from monocytes in the bone marrow and long-lived macrophages that self-renew in tissues [14] (Figure 1). Macrophages can be classified into several subgroups based on their anatomical locations and functional phenotypes, including microglia in the central nervous system (CNS), osteoclasts in bones, alveolar macrophages in the lungs, histiocytes in the spleen and interstitial connective tissues, Kupffer cells in the liver, etc. [14]. In a disease state, both resident macrophages and bone marrow-derived macrophages may play a role, depending on the location where the disease occurs [15,16,17,18].

Here, we describe macrophages based on the expression of cell surface markers, the production of specific factors, and their biological activities. These include classically activated or inflammatory macrophages (M1) and selectively activated or anti-inflammatory macrophages (M2), a process known as “macrophage polarization” [19]. (Table 2 describes macrophages with different phenotypes.) M1 macrophages are typically identified by surface markers like CD80 and CD86. These markers activate their oxidase system, leading to the generation of reactive oxygen species [20,21]. Additionally, M1 macrophages secrete a significant amount of pro-inflammatory cytokines such as IL-1β and TNF-α. These macrophages play the function of antigen presentation, participate in pathogen clearance during infection, and play the role of pro-inflammation, the removal of pathogenic microorganisms, and anti-tumor [22]. Correspondingly, M2 macrophages play an anti-inflammatory function, mainly producing anti-inflammatory factors, such as IL-10, TGF-β, and Arg1, to promote tissue repair and wound healing and promote angiogenesis and fibrosis. In general, M2 macrophages can inhibit inflammation; coordinate and promote tissue remodeling, angiogenesis, and immune regulation; and promote the development of tumors [23].

**Figure 2:**
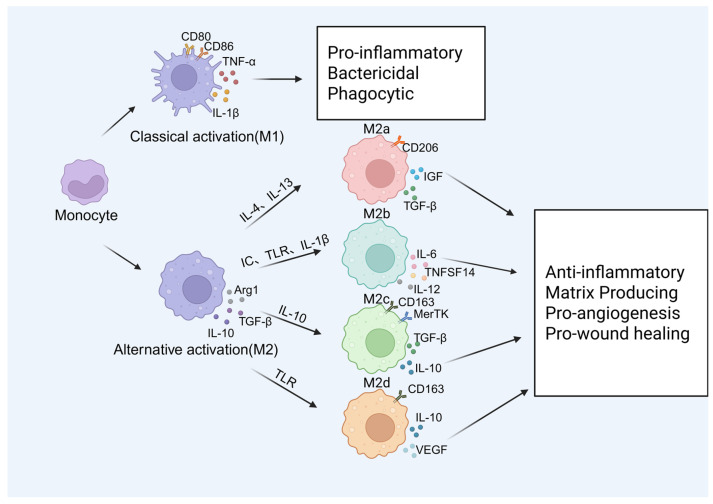
The types of macrophages. Monocyte-derived macrophages can be divided into two types: one is the classical activated M1 type, and the other is the alternative activated M2 type, where M2 can be divided into M2a, M2b, M2c, and M2d.

However, the M1/M2 phenotype does not completely capture the various phenotypic subsets of macrophages. Based on the activation stimulus they receive, M2 macrophages can be classified into four distinct subgroups: M2a, M2b, M2c, and M2d. M2a macrophages express CD206 and HLA-DR while secreting factors such as IL-10 and TGF-β that promote tissue repair [24]. M2b macrophages exhibit low expression of CD163 and HLA-DR. They release cytokines with immunomodulatory effects, including IL-1β, IL-6, TNF-α, CCL1, TNFSF14, and IL-12, under the influence of immune complexes (ICs), Toll-like receptor (TLR) ligands, and IL-1β. M2c macrophages are induced by glucocorticoids, IL-10, and tyrosine kinase. They display high expression of MerRK and exhibit strong anti-inflammatory activity against apoptotic cells [25]. M2d macrophages represent a tumor-associated macrophage subtype that primarily expresses CD163, along with VEGF, IL-10, and TGF-β. They are induced by TLR agonists through adenosine receptors. Upon activation, these adenosine receptors inhibit the production of pro-inflammatory cytokines while promoting the secretion of anti-inflammatory cytokines (high IL-10 and low IL-12) and vascular endothelial growth factor (VEGF) [23]. These factors have been implicated in angiogenesis and tumor growth (Figure 2).

Finally, with the in-depth development of single-cell sequencing and spatial transcriptomics research, macrophages are no longer simply defined as a few subtypes. The new functional classification has gradually become a hot topic of people’s attention [26].

**Table 2:** Different phenotypic markers and secreted factors of M1 and M2 macrophages [23,27,28].

Phenotypes	Markers	Cytokines and Chemokines	Functions
M1	CD80, CD86, CD68, MHCⅡ, TLR2/4, IL-1R, CD163 ^lo*^, and CD40 ^hi*^	TNF-α, IL-1α, IL-1β, IL-6, IL-12, IL-23, COX-2, and iNOS	Promote the Th1 response and the inflammatory response
M2a	CD206 ^hi^, IL-1Rα, IL-1RⅡ, CD163 ^lo^, HLA-DR ^hi^, Arg-1, FIZZ1, and Ym1/2	IL-10, TGF-β, CCL17/18, and CXCL13	Anti-inflammatory and maintain tissue homeostasis
M2b	CD163 ^lo^, HLA-DR ^lo^, and CD86	IL-10 ^hi^, IL-12 ^lo^, IL-1β, IL-6, and TNF-α	Promote the Th2 response
M2c	CD86 ^lo^, CD163 ^hi^, TLR1/8, and MerTK	IL-10, TGF-β, CCL16/18, and CXCL13	Phagocyte apoptotic cells
M2d	CD86 ^lo^, CD163 ^hi^, VEGF, IL-10, and TGF-β	VEGF, IL-10 ^hi^, and IL-12 ^lo^	Angiogenesis and tumor progression

hi*: High expression; lo*: Low expression.

## 3. Structure of Netrin-1 and Its Receptors

**Figure 3:**
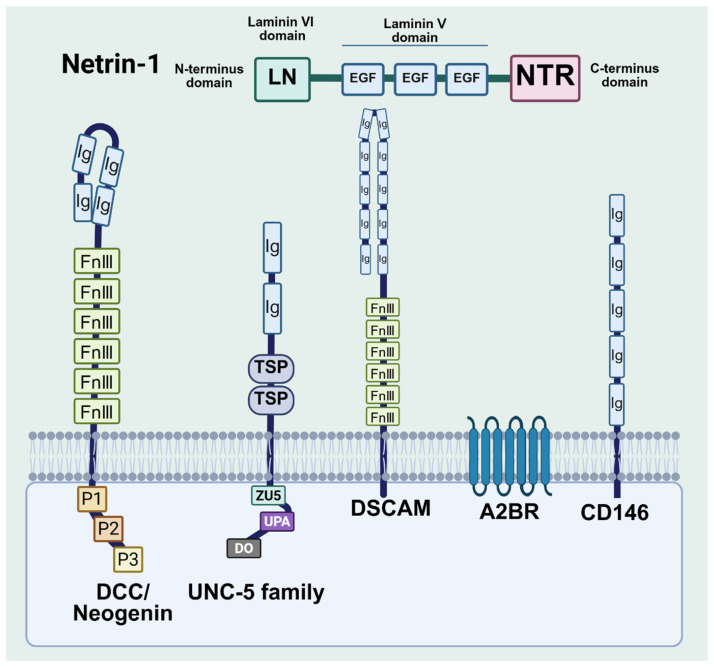
The structure of netrin-1 and its receptors. Netrin-1 is primarily composed of the N-terminal, the C-terminal, and the middle V and VI domains. The receptors associated with netrin-1 include dependent receptors such as DCC, Neogenin, and UNC5b, as well as independent receptors like DSCAM and CD146.

Netrin-1 is a highly conserved, laminin-related molecule encoded by the *NTN1* gene located on chromosome 17. It is composed of 604 amino acids and features a highly conserved N-terminal laminin domain (LN, also known as domain VI). Additionally, domain V contains three cysteine-rich LN-type epidermal growth factor (EGF)-like repeats (LE1, LE2, and LE3), along with a small, positively charged C-terminal domain, referred to as the netrin-like (NTR) module [29]. Domains V and VI are crucial for binding to members of the DCC (deleted in colorectal cancer), Neogenin, and UNC5 families [30] (Figure 3). The functional significance of the NTR module in netrin-1 is largely unknown. The NTR domain is not required for secretion [31], nor receptor binding [32,33,34], but changes in the NTR domain affect the binding capacity of a ligand to its receptors and may also be involved in controlling the activity of metalloproteinases [35]. Its structural disruption may even prevent the presence of secreted reticuloprotein-1 in the extracellular space.

Previous structural studies have identified three independent receptor binding sites on the N-terminal of laminin and three EGF domains of netrin-1 [32,33,34]. These sites serve as platforms for netrin-1 to interact with various receptors, initiating signals that influence axon navigation trajectories and cell fate. Depending on the type of receptor expressed by the target cells, netrin-1 can be used as a chemotactic or exclusionary agent for neuronal cell migration and axonal extension. Cells expressing the netrin-1 receptor DCC or Neogenin are attracted by netrin-1. Conversely, cells expressing the UNC receptor family, such as UNC5, will be rejected by netrin-1 [36]. These netrin-1-dependent receptors belong to the immunoglobulin (Ig) superfamily of proteins. DCC is structured with four N-terminal Ig-like domains arranged in a horseshoe conformation [37], followed by six fibronectin (FN) type III domains, a transmembrane segment, and a large cytoplasmic segment that contains three highly conserved sequence moieties called P1, P2, and P3 [38,39]. The absence of DCC in colon cancer cells is associated with the progression and malignancy of the disease. Netrin-1 binds to DCC at the membrane-proximal FN domains, specifically FN4 and FN5-FN6. Upon binding, a complex is formed, known as the netrin-1/DCC signaling complex, which triggers the homomerization of DCC through the cytoplasmic P3 motif [40]. The process is linked to the chemical attraction of axons [41,42] and recruits an intracellular signaling complex that results in calcium release [43], kinase activation [44], and cytoskeletal rearrangement [45]. In the presence of UNC5, a ternary complex that includes netrin-1 and the extracellular portions of both UNC5 and DCC is formed [32]. Heterodimerization also occurs through cytoplasmic interactions between the P1 motif of DCC and the DCC-binding motif of UNC5, leading to another signaling complex known as netrin-1-mediated heterodimerization of DCC-UNC5, which reverses the axon’s response to a rejection signal [32]. The Ig1-Ig2 tandem repeats of UNC5b play a crucial role in binding netrin-1 [46], with the Ig1 domain of UNC5b being the primary interaction site for netrin-1 [34]. Furthermore, when netrin-1 is depleted in migrating cells and DCC aggregation is prevented, apoptosis may occur [47].

Recently, the Down syndrome cell adhesion molecule (DSCAM) [48] and CD146 (also known as MCAM or Muc18) have been identified as receptors for netrin-1, both of which belong to the immunoglobulin superfamily. CD146 is an angiogenic mediator expressed by endothelial cells; its interaction with netrin-1 is involved in processes such as angiogenesis, vascular permeability, and the trans-endothelial migration of leukocytes, which counteract the anti-angiogenic signal of UNC5b [49]. Other netrin-1 receptors that do not fall under the immunoglobulin superfamily include heparin sulfate proteoglycans, α6β4 and α3β1 integrins, and the adenosine receptor (A2BR) [50]. Netrin-1 mediates the adhesion and migration of epithelial cells through integrins α6β4 and α3β1. It is also involved in the purinergic pathway, binding to A2BR to increase intracellular cAMP levels, thereby enhancing endothelial barrier function and macrophages polarization [1].

In recent years, researchers have discovered that netrin-1 plays a dual role in the immune system during the inflammatory processes. It induces leukocyte chemotaxis through Neogenin while simultaneously inhibiting leukocyte chemotaxis through the UNC5b [51]. This dual action helps alleviate tissue damage, inhibit cell apoptosis, and limit inflammation. Additionally, netrin-1 has been classified as an oncogene in tumorigenesis, as it inhibits macrophages recruitment, promotes cell survival, and stimulates invasive and pathological angiogenesis [52].

## 4. Acute Inflammation

### 4.1. Acute Ischemic Stroke

Acute ischemic stroke occurs as a sudden focal injury to the central nervous system (CNS) due to vascular events, which can include cerebral infarction, intracerebral hemorrhage (ICH), and subarachnoid hemorrhage (SAH) [53]. Following an ischemic stroke, the blood supply to the brain becomes critically insufficient, leading to a lack of oxygen and ultimately resulting in neuronal death [54]. The inflammatory response at the cerebral capillary endothelial interface affects the ischemic tissue. Currently, effective methods to improve functional recovery after a stroke are lacking [55].

The blood–brain barrier is a highly selective structural and functional barrier that separates the blood from the central nervous system. It plays a crucial role in maintaining normal brain function and homeostasis in vivo. Within the neurovascular unit, both nerve cells and non-nerve cells interact with one another [56]. In the case of ischemic stroke, effectively removing tissue debris can aid in tissue reconstruction and the reorganization of neural networks to some extent [57]. Microglia, the primary heterogeneous immune cells in the brain, play an essential role in this process. M1 macrophages are believed to be neurotoxic and contribute to the production of pro-inflammatory cytokines. In contrast, M2 macrophages promote tissue repair and aid in stroke recovery [58] by facilitating phagocytosis and the clearance of neuronal debris, potentially reducing brain damage after a stroke [59]. Depleting microglia can worsen ischemic injury and disrupt neural network activity [60]. Therefore, targeting the activation of phagocytic microglia may effectively inhibit inflammatory damage and support improved recovery after a stroke [61].

Netrin-1 plays a critical role in survival following ischemic events [62]. Previous studies have shown microglial expression of netrin-1 and UNC5a in clinical patients during an ischemic stroke and in rodent models of experimental ischemic stroke [63]. Netrin-1 binds to UNC5a, inhibiting the function of the UNC5 death domain, which ultimately improves the survival rate of microglia under hypoxic conditions. The UNC5 protein acts as a substrate for caspases; its cleavage by caspases can result in apoptotic cell death [64]. Furthermore, both humans and mice microglia show similar adaptations to M2-like microglia to mitigate neuronal damage [65]. The reduced migratory capacity of these cells allows them to effectively manage the local inflammatory microenvironment, mitigate inflammation by limiting the infiltration of inflammatory cells, and promote tissue repair and remodeling [66].

**Figure 4:**
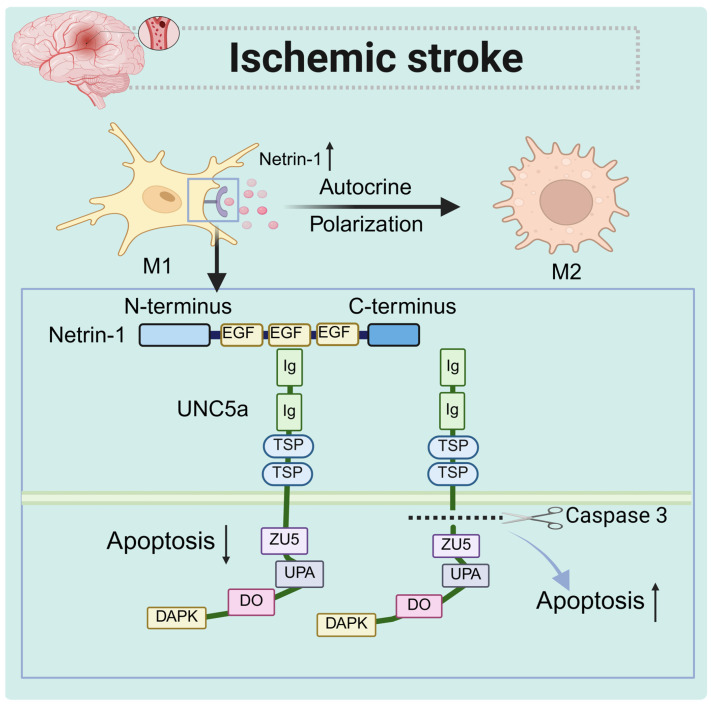
The role of netrin-1 in ischemic stroke. Netrin-1 expressed by macrophages inhibits their own apoptosis by binding to the receptor UNC5a while promoting their own M2 polarization and repairing tissue damage.

In conclusion, netrin-1 in microglia plays a crucial role in immunity by inhibiting apoptosis and promoting cell survival through its binding with UNC5a. Additionally, microglia that express netrin-1 are more likely to be polarized toward the M2 type, which enhances their anti-inflammatory and repair functions (Figure 4).

### 4.2. Acute Lung Injury (ALI)

Acute lung injury (ALI) is a clinical syndrome characterized by damage to lung tissues, resulting in a variety of pathological and structural changes. Key features of ALI include alveolar injury, the development of pulmonary edema, inflammation driven by eosinophils, and the dysfunction of surface-active substances [67,68]. During the inflammatory process associated with ALI, cytokines such as IL-8, IL-1β, IL-36, and CXCL2 play crucial roles in mediating the aggregation and infiltration of various immune cells into the lungs. This activation triggers intracellular signaling pathways and leads to the release of large quantities of cytokines. As immune cells become continuously activated, a vicious cycle is established, ultimately resulting in a cytokine storm [69].

During the early stages of inflammation, monocytes are recruited to the affected area, where they differentiate into macrophages and secrete relevant inflammatory cytokines. Studies have shown that the expression of netrin-1 is reduced in lung tissues [70]. In cases of lung injury induced by lipopolysaccharides (LPSs), netrin-1 has been found to decrease the levels of Toll-like receptor 4 (TLR4) and prevent the nuclear translocation of nuclear factor-κB (NF-κB). When inflammation occurs, NF-κB transcription is activated, while the transcription of netrin-1 is inhibited [71]. The A2BR on neutrophils can bind to netrin-1, which limits neutrophil infiltration at the site of acute inflammatory lung injury [72]. Consequently, the downregulation of netrin-1 expression is associated with increased recruitment of neutrophils to areas of inflammation.

**Figure 5:**
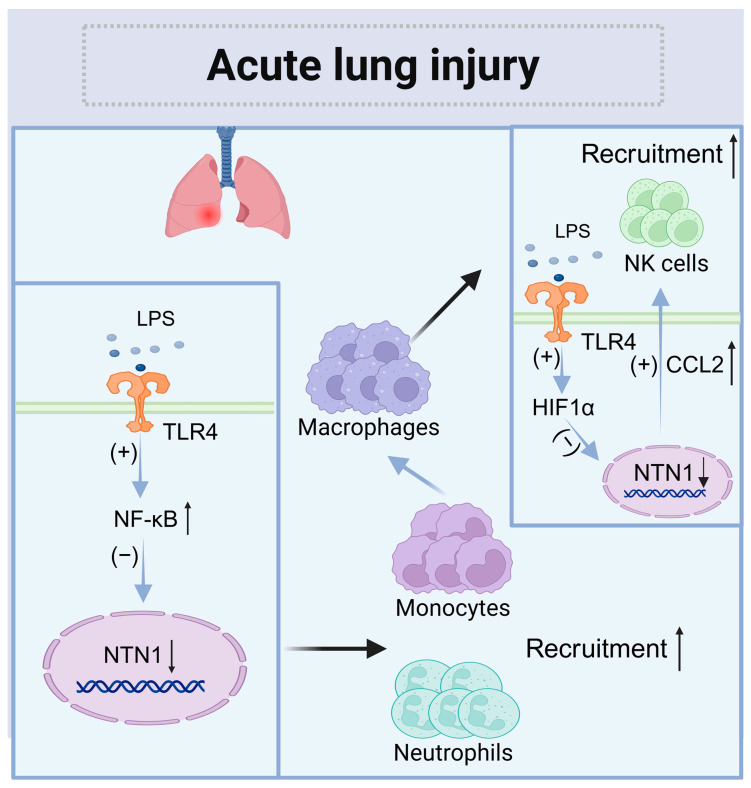
The role of netrin-1 in acute lung injury. When local inflammation occurs, the NF-κB pathway is activated, inhibiting the transcription of *NTN1* and increasing the recruitment of monocytes and neutrophils. Meanwhile, the expression of CCL2 in macrophages increased, and NK cells were recruited. (↑: increased; ↓: decreased.)

Furthermore, during the early stages of LPS-induced lung injury in mice, the expression of netrin-1 was found to be upregulated in myeloid cells. Myeloid cells, such as macrophages and neutrophils, play a crucial role in innate immunity and are key drivers of both the initiation and resolution of inflammation. Netrin-1 expressed by macrophages can regulate CCL2 secretion, thereby limiting NK cell recruitment [73]. NK infiltration was increased after the depletion of netrin-1 in myeloid cells. Additionally, depleting NK cells in mice has been shown to decrease chemokine-mediated neutrophil recruitment [73]. Furthermore, treating macrophages ex vivo or promoting endogenous overexpression of netrin-1 in vivo can shift macrophages toward an anti-inflammatory M2-like phenotype. This shift limits inflammatory progression and promotes wound healing [74]. Overall, the supplementation of exogenous netrin-1 may represent a promising future therapeutic option (Figure 5).

### 4.3. Acute Kidney Injury (AKI)

Acute kidney injury (AKI) is a condition marked by a sudden decrease in kidney function, which is linked to high mortality rates, impaired organ performance, and the potential development of chronic kidney disease [75]. One key mechanism involved in AKI is ischemia–reperfusion [76]. When the blood supply returns to the kidney after a period of reduced flow, it triggers a strong inflammatory response, leading to increased oxidative stress in the damaged kidney [77].

When inflammation occurs, netrin-1 expression is decreased, renal injury and apoptosis are increased, monocyte and neutrophil infiltration are increased, and the production of cytokines (IL-6, IL-1β, and TNF-α) and chemokines is increased [78]. The activation of NF-κB occurs during inflammation; NF-κB is a well-known regulator of the cyclooxygenase pathway. Netrin-1 influences the expression of COX-2 by modulating NF-κB activation. Inhibiting COX-2-mediated production of PGE2 helps to regulate inflammatory responses in neutrophils and macrophages. The infiltration of neutrophils and monocytes is a marker of tissue damage in renal ischemia–reperfusion injury. Increased COX-2 expression results in higher PGE2 production, which subsequently enhances IL-23-mediated migration of neutrophils by increasing IL-17 production [79]. Additionally, netrin-1 binds to UNC5b on neutrophils and monocytes, inhibiting their migration and the release of inflammatory factors. When UNC5b is neutralized, there are increases in monocyte and neutrophil infiltration, along with higher production of serum and renal cytokines and chemokines, ultimately resulting in greater renal injury and tubular cell apoptosis [80].

**Figure 6:**
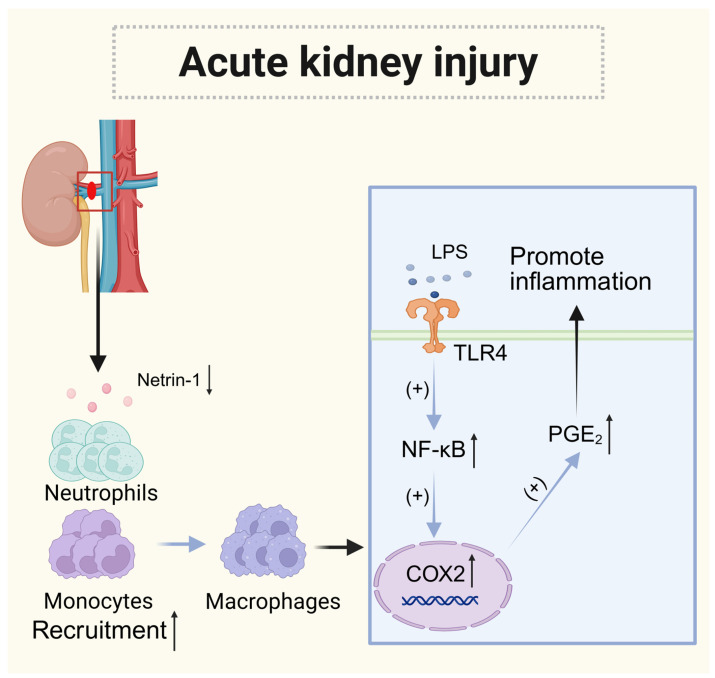
The role of netrin-1 in acute kidney injury. During acute kidney injury, the expression of netrin-1 is downregulated, and the recruitment of monocytes and neutrophils increases. Meanwhile, netrin-1 increases the expression of COX-2 by regulating the activation of NF-κB, and the increase in COX-2 expression leads to an increase in the production of PGE2. Inhibiting the production of PGE2 mediated by COX-2 helps regulate the inflammatory responses of neutrophils and macrophages.

In addition, the overexpression of netrin-1 has been shown to inhibit IFNγ-induced M1 polarization and the production of inflammatory mediators, such as IL-6 and IP-10. It also promotes macrophage expression of Arg-1, IL-4, and IL-13 while decreasing COX-2 expression in the kidney. This leads to a phenotypic shift in macrophage polarization toward an M2-like phenotype [81]. Therefore, netrin-1 may represent a promising therapeutic option for the treatment of renal ischemia–reperfusion injury (Figure 6).

## 5. Chronic Inflammation

### 5.1. Diabetes

Diabetes is a chronic inflammatory disease characterized by consistently high blood glucose levels, which result from defective insulin secretion or impaired insulin action [82]. The process of wound healing in individuals with diabetes is complex and involves four key phases: hemostasis, inflammation, proliferation, and remodeling [83]. In diabetic wounds, the body’s internal environment is disrupted due to persistent hyperglycemia. This dysregulation results in a prolonged inflammatory phase and impaired vascularization, which hinder proper healing [84,85].

Netrin-1 is highly expressed during the inflammatory and proliferative phases of healing. It can bind to the A2BR on macrophages, activating the STAT/PPARγ signaling pathway and regulating the M2 conversion of macrophages, which is the main process in wound healing. Additionally, netrin-1 works synergistically with endothelial cells to promote vascular regeneration [86] and aids in wound healing through macrophage-secreted factors like TGF-β, IGF-1, and VEGF. However, a sustained hyperglycemic environment can impair macrophage functions, leading to decreased secretion of netrin-1 and inhibiting the phenotypic transformation of macrophages. This persistent inflammatory state can delay wound healing. Therefore, exogenous supplementation of netrin-1 is a potential treatment option for this condition. Gelatin methacrylate (GelMA) is a photopolymerizable and injectable hydrogel [87,88,89] that can be loaded with various active substances. It has an extracellular matrix-like structure, providing good biocompatibility, biodegradability, and the ability to maintain a moist [84,90,91] environment for wounds. The covalent immobilization of netrin-1 with acrylate-PEG-NHS in the GelMA hydrogel (GelMA-c-netrin-1) helps maintain their natural bioactivity and allows for long-term release [52]. Meanwhile, exosome transplantation has also emerged as a promising therapeutic approach. Diabetic limb ischemia (DLI) is a common complication of diabetes mellitus, characterized by reduced blood flow to the lower extremities. This condition can lead to chronic pain, non-healing ulcers, and, in severe cases, limb amputation [92]. Netrin-1-enriched exosomes (N-Exos) can mediate endothelial cell proliferation, migration, and angiogenesis via the PI3K/AKT/eNOS and MEK/ERK pathways. Furthermore, N-Exos promote the polarization of macrophages from the M1 to the M2 [93] phenotype, which helps reduce inflammation.

**Figure 7:**
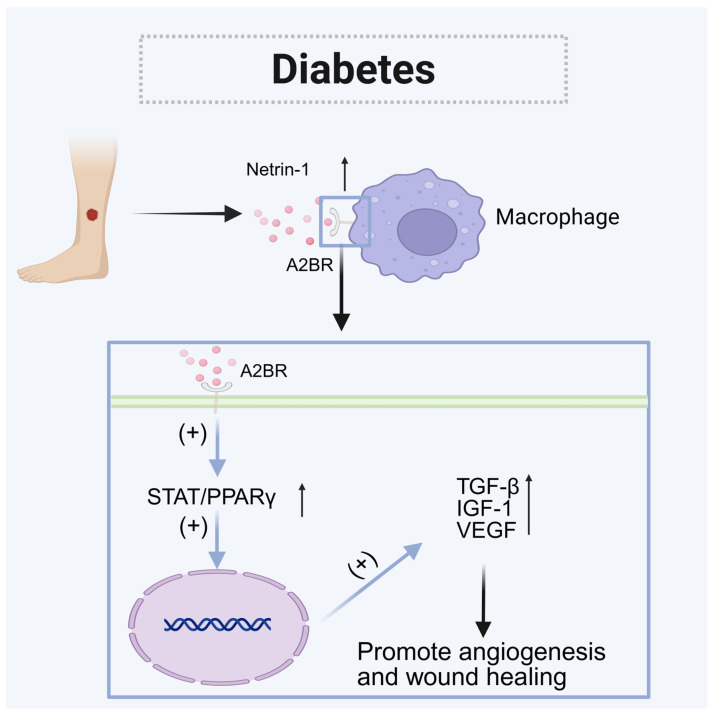
The role of netrin-1 in diabetes. Netrin-1 binds to A2BR on macrophages, activates the STAT/PPARγ signaling pathway, and regulates M2 transformation in macrophages. And through factors such as TGF-β, IGF-1, and VEGF secreted by macrophages, it works synergistically with endothelial cells to promote vascular regeneration and wound healing.

Exosome therapy and hydrogel biomolecular delivery provide effective alternatives for the treatment of diabetic wound healing and lower limb ischemia, which can effectively address the limitations of traditional medical and surgical approaches (Figure 7).

### 5.2. Coronary Artery Disease (CAD)

Coronary artery disease is a prevalent form of heart disease that causes myocardial ischemia mainly due to the hardening, narrowing, or blockage of the coronary arteries. This condition is marked by the buildup of lipids and immune cells in the walls of the arteries and is often associated with atherosclerosis [94]. Atherosclerosis frequently affects middle-aged and elderly individuals, primarily targeting the larger and medium-sized arteries, which eventually leads to the narrowing of the artery lumen and can result in complete blockage. The rupture of unstable atherosclerotic plaques, along with platelet aggregation and thrombosis, can cause vascular stenosis or occlusion, culminating in acute cardiovascular diseases [95].

Endothelial cells (ECs) serve as a blood vessel barrier, forming a semi-permeable monolayer that separates the artery wall from the blood flow components within the vessel. This barrier plays a crucial role in regulating blood vessel tone, preventing platelet aggregation, and maintaining fluid balance. Studies have shown that the expression of netrin-1 in the vascular lumen is higher in healthy humans, while the expression of netrin-1 in the vascular lumen is decreased in atherosclerosis [96,97]. Netrin-1 inhibits monocyte adhesion and migration by blocking the NF-κB pathway, which reduces the expression of CCL2, IL-6, and ICAM-1. It also possesses anti-inflammatory properties and helps maintain endothelial barrier function.

However, in patients with CAD, lipid-phagocytic macrophages exhibit higher levels of intracellular netrin-1, which demonstrates greater accumulation of these macrophages in vivo. Both netrin-1 and UNC5b have been observed in atherosclerotic plaques in mice and humans, particularly within foam cells. Their presence is induced by hypoxia and the accumulation of oxidized low-density lipoprotein (ox-LDL) [98]. Hypoxic stress is closely associated with atherosclerosis and activates inflammatory transcription factors in foam cells [99]. During this process, oxLDL binds to the specific receptor CD36 [100] to activate the transcription factor NF-κB [101,102]. Hypoxia-inducible factors [103], such as HIF1-α and NF-κB, regulate the transcription of netrin-1 and UNC5b [104]. Recombinant netrin-1, as well as netrin-1 secreted by foam cells formed in vitro, can effectively block the directed migration of macrophages to CCL19 [105]. Furthermore, netrin-1 inhibits migration by disrupting Rac1 signaling pathways, reorganizing the actin cytoskeleton, and affecting cell polarization. In the context of atherosclerotic plaques, netrin-1 may serve to anchor foam cells, preventing their migration into the lumen or lymphatic system. Moreover, during atherosclerosis development, netrin-1 binds to Neogenin in the inner and lower smooth muscle cells (SMCs), promoting their migration to the plaques and contributing to the progression of the disease [106,107].

A family without hyperlipidemia but with a history of early-onset CAD underwent exome sequencing, which did not reveal any known mutations in genes linked to atherosclerosis. However, a single-nucleotide polymorphism (SNP) was identified in the *NTN1* gene [107]. This SNP results in a single-amino-acid substitution, specifically Arg590Leu, within the highly conserved NTR domain of netrin-1, a protein involved in axon guidance [108]. Despite this mutation being located outside the known binding sites of netrin-1, it had a significant impact on the protein’s receptor-binding properties. The Arg590Leu mutation decreased netrin-1’s binding to UNC5b, DCC, and integrin-β3 while simultaneously increasing its affinity for Neogenin and heparin sulfate. Functional analyses demonstrated that these alterations in the binding interactions of netrin-1 led to a reduced ability of the mutated protein to diminish endothelial cell activation and monocyte adhesion. In vitro migration experiments revealed that the mutant netrin-1 blocked chemokine-directed macrophage migration more effectively than the wild-type protein. Given that the adherence of monocytes to the endothelium and their migration into the intima are critical factors in arterial inflammation, the mutated netrin-1 protein contributes to the accumulation of macrophages within plaques by obstructing their exit. This, coupled with enhanced monocyte infiltration, worsens arterial wall inflammation and promotes plaque progression [109].

**Figure 8:**
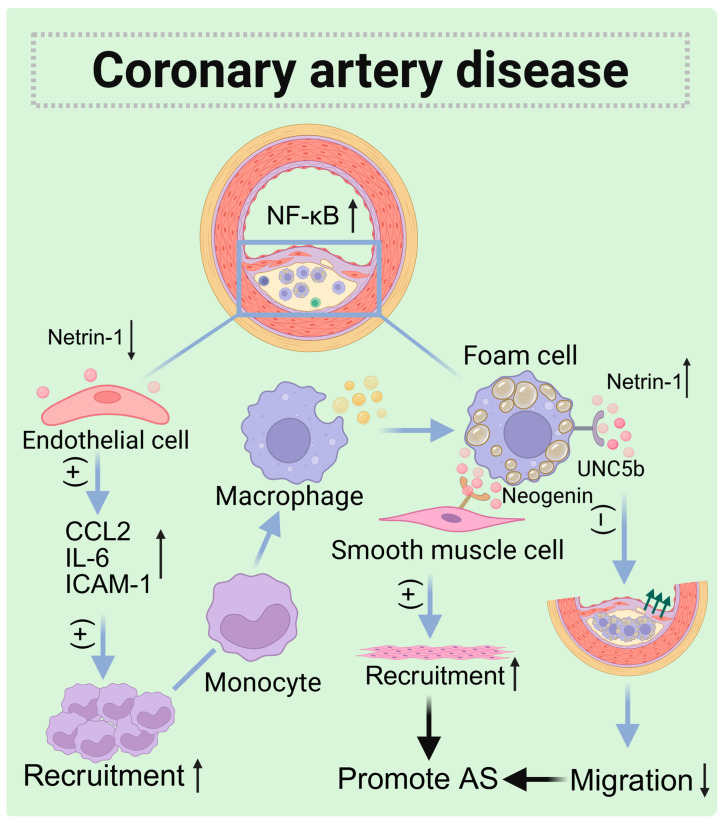
The role of netrin-1 in atherosclerosis. The expression of netrin-1 in endothelial cells within the vascular lumen of patients with atherosclerosis is decreased, and the recruitment of monocytes and neutrophils increases. Macrophages phagocytize lipids to become foam cells that deposit on the vascular wall. At this location, the expression level of netrin-1 in macrophages is relatively high, and it binds to its own UNC5b to inhibit migration. Meanwhile, the Neogenin receptor on smooth muscle cells binds to netrin-1 to synergistically promote the progression of atherosclerosis. (↑: increased; ↓: decreased.)

Netrin-1 exhibits both pro-atherosclerotic and anti-atherosclerotic functions, depending on its source. When produced by circulating endothelial cells, netrin-1 has a protective effect against atherosclerosis. Conversely, netrin-1 generated by plaque-accumulating macrophages has the opposite effect, with both functions mediated by UNC5b. In plaques, netrin-1 not only prevents the excretion of inflammatory cells but also induces the recruitment of smooth muscle cells to the intima through Neogenin, thereby promoting lesion progression [8]. Additionally, mutations in the NTR domain of netrin-1 can disrupt its ability to bind to various receptors, influencing disease progression. In the future, we will be able to target the functional changes resulting from mutations in macrophage-derived netrin-1 at specific amino acid sites. This could lead to targeted drug delivery, result in better control of disease progression, and ultimately aid in the treatment of atherosclerosis (Figure 8).

### 5.3. Abdominal Aortic Aneurysm (AAA)

An abdominal aortic aneurysm (AAA) is a condition that involves the degeneration of the aortic wall, leading to significant vascular damage and progressive structural changes in the abdominal aorta [110]. While there are considerable variations in how the disease progresses and its severity, the early stages of AAAs are typically asymptomatic. When an AAA ruptures, the mortality rate can be as high as 90%, making it a silent killer [111]. To prevent a life-threatening rupture of the blood vessel, surgical intervention remains the primary treatment for this complex disease. Currently, there are no non-invasive treatments available for the early stages of AAAs. Therefore, gaining a better understanding of the biological mechanisms behind the onset and progression of AAAs is crucial for identifying new diagnostic methods and therapeutic targets, which would provide essential support for developing targeted intervention therapies for this condition.

Studies have found that netrin-1 secreted by macrophages is involved in macrophage-driven damage and extracellular matrix (ECM) degradation [112]. Notably, macrophages that were abundant in netrin-1 also exhibited high levels of pro-inflammatory and pro-angiogenic markers. In contrast, macrophages with lower levels of netrin-1 showed elevated expression of the anti-inflammatory marker mannose receptor 1 (Mrc1) and the anti-atherosclerosis gene [106,113]. Moreover, the absence of macrophage-derived netrin-1 protected mice from developing abdominal aortic aneurysms (AAAs), suggesting that netrin-1 produced by macrophages plays a pathogenic role in the development of AAA.

In an abdominal aortic aneurysm (AAA), chronic activity of the extracellular matrix (ECM)-degrading enzyme matrix metalloproteinases (MMPs) has consistently been shown to significantly impair vascular remodeling, often leading to aortic rupture [114]. Specifically, the absence of MMP3 has been found to reduce ECM damage and lower the susceptibility to an AAA in mice. Macrophages are often associated with MMP activity. Macrophage-derived netrin-1 plays a crucial role in regulating the transcription of matrix metalloproteinase-3 (MMP3) and calcium mobilization in neighboring vascular smooth muscle cells (VSMCs) through Neogenin. The activation of Neogenin by netrin-1 is essential for the nuclear translocation of the T-cell transcription factor NFATc3, which enhances the catalytic activity of MMP3. Furthermore, mechanical load on the blood vessel wall is a pathological marker of life-threatening AAAs. VSMCs directly activate the mechanosensory ion channel Piezo1 by up-regulating the cytoskeletal crosslinking agent α-actinin2 in the presence of netrin-1, leading to the formation of a solid-like mechanical state [115]. Piezo1 acts as a mechanical sensor on the cell membrane, which, when activated by mechanical forces, allows ions such as Ca^2+^ to enter the cell [116,117]. Netrin-1 is released from macrophages that infiltrate the vessel wall and contributes to AAAs by sustaining downstream Ca^2+^ signaling necessary for stimulating the matrix degradation of MMP3 in VSMCs [112].

**Figure 9:**
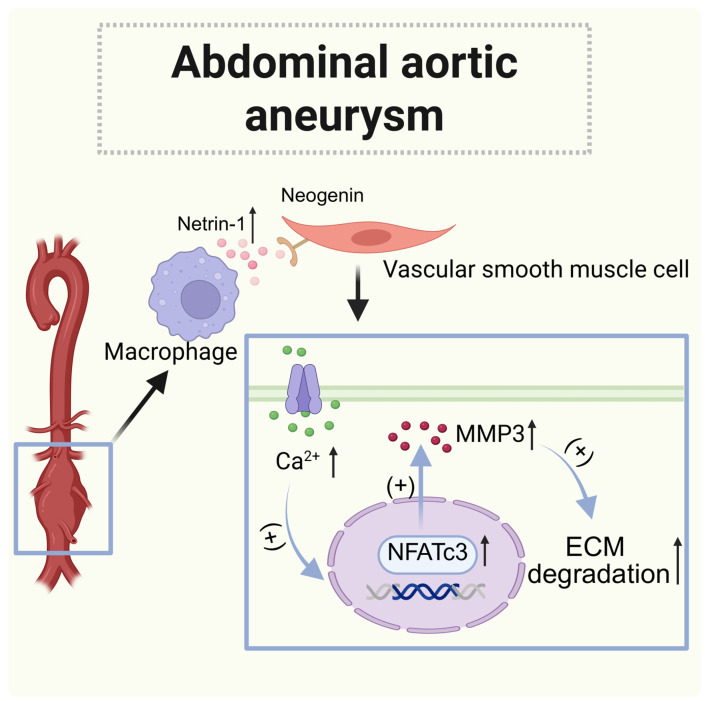
The role of netrin-1 in abdominal aortic aneurysms. The expression of netrin-1 in macrophages is increased; when bound to the Neogenin receptor on vascular smooth muscle cells, it promoted Ca influx, increased the expression of MMP3, and increased ECM degradation. (↑: increased.)

Finally, at the therapeutic level, metformin can inhibit the phenotypic transformation of VSMCs mediated by macrophages, reduce the incidence of AAAs, and help prevent the progression of AAAs in patients with diabetes. Therefore, the development of metformin-supported netrin-1-responsive nanoparticles (Tgt-NP-Met) may contribute to the targeted therapy of AAAs [118] (Figure 9).

### 5.4. Osteoarthritis(OA)

Osteoarthritis is a chronic joint disease characterized by structural abnormalities and a functional decline in the synovial joints [119]. The primary reason patients seek medical treatment for osteoarthritis is pain, with subchondral bone lesions being a significant source of this pain.

Abnormal remodeling of subchondral bone contributes to the degeneration of articular cartilage [120]. During the early stages of osteoarthritis (OA), the number of osteoclasts increases, leading to preosteoclast-induced angiogenesis [121]. Elevated osteoclast activity stimulates the production of excess transforming growth factor beta 1 (TGF-β1), which recruits bone marrow mesenchymal stem cells, resulting in abnormal subchondral bone formation. The administration of TGF-β1-neutralizing antibodies can help slow the progression of OA by targeting the pathological features of subchondral bone [122]. The osteochondral junction has long been recognized as an early site for the development of new blood vessels. Perivascular sensory and sympathetic nerve fibers can disrupt the integrity of the osteochondral junction [123]. Subchondral bone remodeling initiated by osteoclasts can contribute to OA pain, possibly through the secretion of netrin-1 by osteoclasts. Netrin-1 is found on the membranes of osteoclasts in fluid from OA patients, as well as in mouse models of the temporomandibular joint (TMJ) [124]. Netrin-1 not only acts as a strong stimulant for blood vessel growth but also promotes the proliferation, migration, and adhesion of vascular endothelial cells and smooth muscle cells, playing a significant role in angiogenesis and inflammation [125]. Increased levels of netrin-1 secreted by osteoclasts in OA have been linked to the autocrine and paracrine secretion of UNC5b, which promotes osteoclast differentiation [126]. Netrin-1 enhances this differentiation by binding to its UNC5b. Additionally, netrin-1 interacts with the DCC on sensory neurons. This interaction activates the downstream PI3K/AKT [127] pathway, which induces axonal growth and sensory innervation in the subchondral area [128]. Mice lacking netrin-1 experienced milder OA pain, suggesting that inhibiting netrin-1 or DCC in osteoclasts can alleviate osteoarthritis-related pain [129].

**Figure 10:**
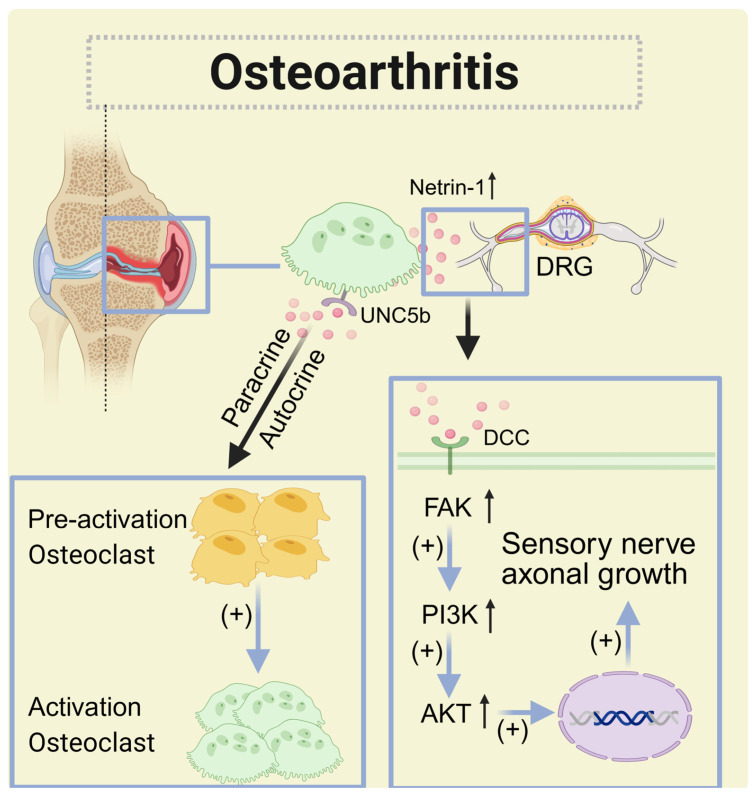
The role of netrin-1 in osteoarthritis. The netrin-1 secreted by osteoclasts binds to UNC5b to promote self-activation and proliferation and at the same time binds to DCC on DRG neurons to promote the growth of sensory nerves. (↑: increased.)

Netrin-1, secreted by osteoclasts, promotes neurite growth primarily through DCC. Targeting axon-guiding molecules like netrin-1 from abnormal subchondral bone remodeling may offer potential treatments for OA pain (Figure 10).

### 5.5. Pulmonary Fibrosis

Idiopathic pulmonary fibrosis (IPF) is a chronic, progressive respiratory disease that typically affects older adults [130]. It is characterized by the gradual scarring of lung tissue, which results in decreased gas exchange and ultimately leads to respiratory failure [131]. Key features of IPF include the proliferation of myofibroblasts, collagen buildup, and damage to the alveolar epithelial cells [132]. The development of fibrosis is linked to prolonged or recurrent injury to the epithelial tissue, which activates fibroblasts through mechanisms that are not yet fully understood. It is currently believed that this process is coordinated by macrophages to varying degrees [133].

Myofibroblasts and macrophages can create a spatially restricted fibrotic niche [134]. Proteins that guide neurons, such as netrin-1, promote inflammatory scarring. The expression of netrin-1 in IPF is increased. The deletion of netrin-1 derived from macrophages affects collagen accumulation, fibrotic histology, and neuro-related functions in IPF. The IPF lung tissue contains a high concentration of netrin-1^+^ macrophages and norepinephrine. Netrin-1 produced by macrophages promotes adrenergic neural development and the repair of sensory nerves [135] by interacting with DCC. This interaction reshapes adrenergic nerves and increases the level of norepinephrine required to stimulate fibrosis [136]. Treatment with α1 adrenergic receptor blockers can improve the survival rate.

**Figure 11:**
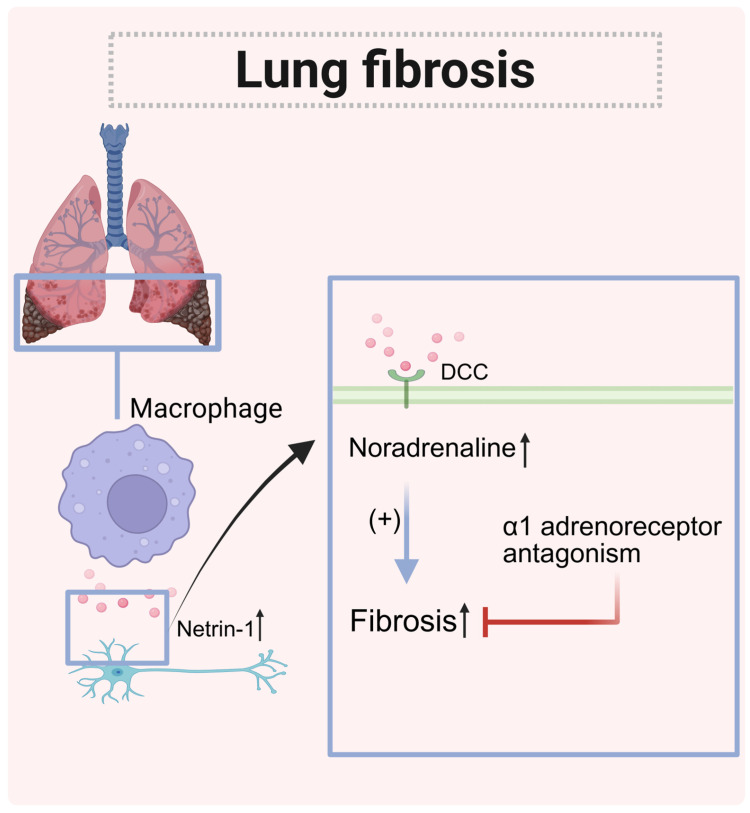
The role of netrin-1 in lung fibrosis. The netrin-1 expressed by macrophages binds to the receptor DCC on neurons to promote the expression of norepinephrine, thereby promoting fibrosis. (↑: increased.)

Since netrin-1 derived from macrophages has a regulatory effect on pulmonary fibrosis, netrin-1 precisely targeting macrophages may become a new therapy with better efficacy than adrenergic antagonists in the treatment of IPF and related diseases in the future [134] (Figure 11).

### 5.6. Endometriosis

Endometriosis is a chronic, estrogen-dependent, inflammatory gynecological condition in which endometrial tissue grows outside the uterus [137]. The cause of endometriosis is believed to be retrograde menstruation, a process where fragments of the endometrium are expelled into the abdominal cavity through the fallopian tubes. These fragments can then interact with peritoneal structures and evade the pelvic immune system, allowing them to attach, invade, establish blood vessels, and form endometriosis lesions [138]. Endometriosis is the most common cause of chronic pelvic pain in women of reproductive age [139]. Processes such as angiogenesis and neurogenesis are believed to contribute to the development of endometrial debris into endometriosis, leading to subsequent pelvic pain [140].

Studies indicate that serum levels of netrin-1 are significantly elevated in endometriosis patients and are positively correlated with pain symptoms [141]. The local accumulation of netrin-1 produced by macrophages promotes the occurrence and development of endometriosis by regulating the proliferation, tubule formation, migration, and invasion of vascular endothelial cells and inducing neuroangiogenesis [141]. The depletion of macrophages led to a reduction in lesion size and blood vessel formation [142]. The amount of vascular endothelial growth factor (VEGF) produced by peritoneal macrophages increases in women with endometriosis. The netrin-1 receptor CD146 is a high-affinity receptor that can activate endothelial cells and initiate downstream VEGF signaling pathways [143]. In the endometriosis microenvironment, the expression of this receptor is significantly increased in vascular endothelial cells. Additionally, Neogenin, another receptor, is overexpressed in sensory neurons that infiltrate endometriosis tissue. The combination of netrin-1 and Neogenin activates the MAPK signaling pathway, leading to an upregulation of MAP4, TAU, and CGRP, which promotes myoduct formation and supports neuronal regeneration [144]. Netrin-1 also enhances the growth and migration of Schwann cells through its interaction with DCC [145] and UNC5b [146], thereby facilitating the regeneration of peripheral nerves.

**Figure 12:**
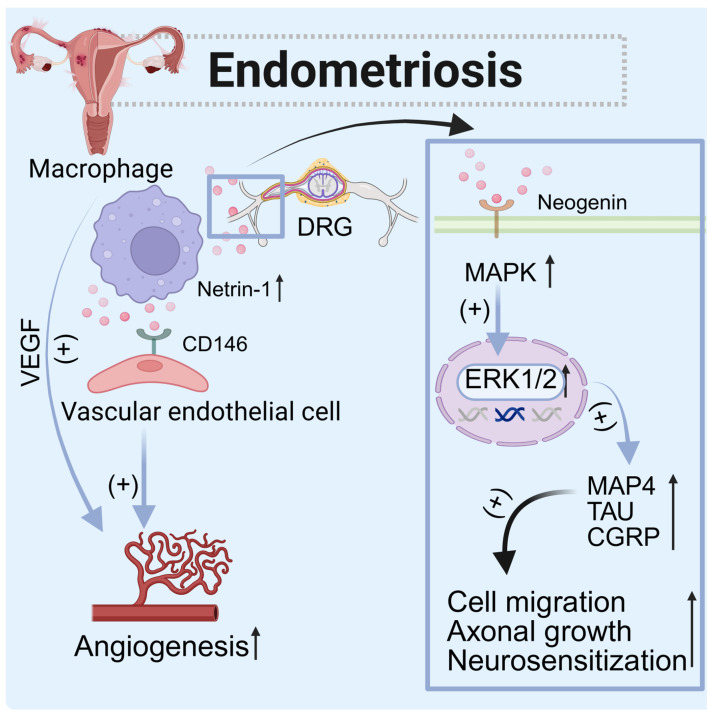
The role of netrin-1 in endometriosis. The netrin-1 secreted by macrophages, on the one hand, binds to CD146 in vascular endothelial cells to promote vascular regeneration, and, on the other hand, binds to Neogenin in DRG neurons to promote cell migration, axonal growth, and neurosensitization. (↑: increased.)

In conclusion, netrin-1 promotes angiogenesis by interacting with vascular endothelial cells, on the one hand, and activates Neogenin to stimulate neuronal regeneration on the other. Additionally, macrophage-derived netrin-1 plays a crucial role in enhancing neuroangiogenesis in ovarian endometriomas. Interventions that disrupt this process may offer promising treatment strategies for endometriosis in the future (Figure 12).

## 6. Cancer

### 6.1. Glioblastoma (GBM)

Glioblastoma (GBM) is the most invasive tumor among gliomas. In the tissue microarray analysis of glioma, *NTN1* is closely related to the poor prognosis of patients. Under normal circumstances, the concentration of netrin-1 in the brain varies between 50 and 150 ng/m. However, under pathological conditions, its expression can increase by 2.1 to 4.5 times [147], exhibiting diffuse expression throughout the tumor. Various cell types within the GBM, including glial cells (astrocytes and oligodendrocytes), microglia, infiltrating immune cells (monocytes, macrophages, and lymphocytes), ECs, and pericytes, can express both netrin-1 and its receptors [135,148,149]. Studies indicate that netrin-1 expression in GBM promotes tumor angiogenesis. The activation of netrin-1/Neogenin may activate integrin β1 (ITGB1) through focal adhesion kinase (FAK), subsequently activating C-MYC in GBM. This activation leads to increased expression of VEGF, which in turn promotes angiogenesis and tumor metastasis [150,151]. Furthermore, C-MYC expression can also be induced through the interaction between netrin-1 and the UNC5a receptor. This interaction can enhance glioma growth by activating C-MYC through the NF-κB pathway [147,152]. During development, netrin-1 plays a role in regulating cell motility and stem cell self-renewal. It can trigger the activation of the DCC/PKC and INα6β4/FAK pathways, which primarily induce the ERK/JNK/NF-kB signaling cascade [153]. This cascade leads to the degradation of E-cadherin via MMP12, resulting in disrupted cell–cell adhesion and increased cell motility. Additionally, netrin-1 enhances the self-renewal of embryonic stem cells by the classic netrin-1 receptor UNC5B [154].

### 6.2. Non-Small Cell Lung Cancer (NSCLC)

Lung cancer is traditionally classified into two main histological subtypes: small cell lung cancer and non-small cell lung cancer (NSCLC). NSCLC is the most prevalent subtype, accounting for approximately 85% of lung cancer cases [155]. Metastasis is a complex multi-step process that involves multiple pathological mechanisms, including epithelial-to-mesenchymal transition (EMT) [156,157]. Moreover, cells exhibiting EMT-like characteristics can acquire the ability to form angiogenic mimicry (VM), which is commonly linked to poor overall survival rates and increased metastasis in many types of cancer [158].

EMT in cancer cells typically enhances their invasive and migratory properties by reducing intercellular adhesion and promoting cell movement. Research has shown that netrin-1 is significantly elevated in NSCLC tissues, and its expression is markedly higher in the tissues of metastatic patients compared to those in the primary tumor [159]. Netrin-1 facilitates EMT through the PI3K/AKT and ERK pathways, which promote the invasion, migration, and VM of tumor cells, thereby enhancing the metastatic potential of NSCLC cells [159,160]. Furthermore, netrin-1 expressed in lung cancer cells increases their survival by inhibiting apoptosis induced by the UNC5a and UNC5b receptors [161].

### 6.3. Other Cancers

**Figure 13:**
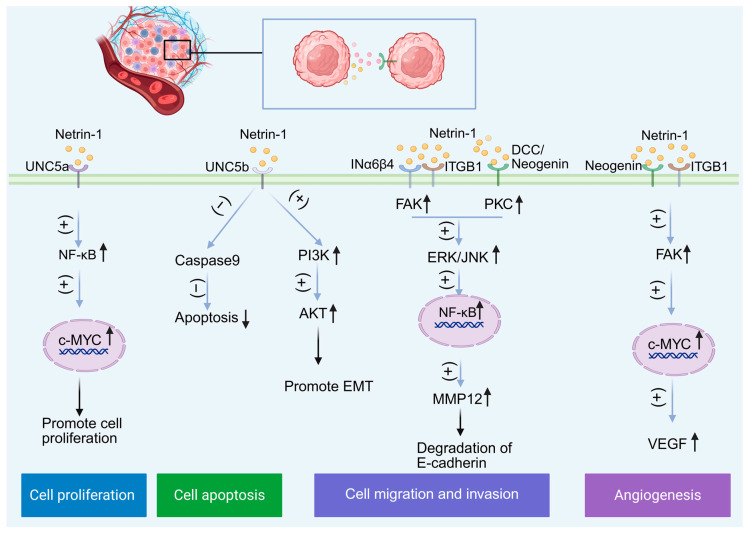
The role of netrin-1 in cancers. Netrin-1 is involved in tumor cell proliferation, metastasis, and invasion, as well as angiogenesis, and promotes tumor progression. (↑: increased; ↓: decreased.)

Numerous studies have reported that netrin-1 exhibits increased expression in tumor cells and the tumor microenvironment, with its levels rising during cancer progression. This suggests that netrin-1 could potentially serve as a predictor of disease outcomes. In the context of inflammation-related colorectal cancer [30], metastatic breast cancer [30,162], bladder cancer [163], and liver cancer [164], netrin-1 appears to play a role in promoting tumor cell growth, invasion, and metastasis, etc. Meanwhile, studies have reported that the netrin-1 monoclonal antibody NP137 in endometrial cancer [4] and cutaneous squamous cell carcinoma [5] can not only block the binding of netrin-1 to UNC5b to directly induce cell apoptosis and inhibit tumor cell proliferation but also effectively control the occurrence of EMT in tumors. Moreover, the combination of NP137 and chemotherapy drugs can not only inhibit the drug resistance of tumor cells but also promote the killing of tumor cells by chemotherapy drugs (Figure 13).

## 7. Conclusions

Netrin-1 has distinct roles in both acute and chronic inflammation. This article examines the various functions of macrophage-derived netrin-1 in inflammatory diseases. Netrin-1, secreted by microglia, regulates their apoptotic capabilities, promotes M2-like transformation, and inhibits apoptosis, all of which aid in tissue repair following a stroke. In acute lung injury, decreased expression of netrin-1 from macrophages facilitates the recruitment of NK cells. Additionally, netrin-1 plays a role in regulating the migration and recruitment of leukocytes during acute inflammation. When netrin-1 levels drop, there is a downregulation of migration inhibitory factors in myeloid cells, such as neutrophils and monocytes, leading to increased infiltration at local inflammatory sites.

In contrast, during the early stages of chronic inflammation, netrin-1 is locally elevated in diabetic wounds and binds to macrophages through A2BR. This interaction promotes angiogenesis and aids in wound healing. However, hyperglycemia significantly decreases the expression of netrin-1, negatively impacting wound healing capabilities. Furthermore, the increased expression of macrophage-derived netrin-1 in the vessel wall of atherosclerosis (AS) prevents the outflow of foam cells, which, unlike netrin-1 from endothelial cells, exacerbates the condition. Netrin-1 secreted by osteoclasts influences the growth of sensory nerve axons, leading to nerve sensitization and pain. Macrophages also play a role in the progression of pulmonary fibrosis through the secretion of netrin-1. Similarly, endometriosis behaves like osteoarthritis, resulting in the overexpression of sensory neurons, which contributes to pain and increased angiogenesis. According to the existing literature, netrin-1 primarily regulates leukocyte migration and infiltration during acute inflammation. In chronic inflammation, however, macrophage-derived netrin-1 levels rise, promoting disease progression and neural sensitization due to the overexpression of neurons. Netrin-1 appears to be a carcinogenic regulator in cancer, inhibiting tumor cell apoptosis and inducing tumor cell growth, migration, invasion, and metastasis.

As netrin-1 is considered to be a potential therapeutic target for cancer and inflammatory diseases, both macrophage-engineered exosomes and hydrogels can target macrophage-derived netrin-1 for treatment. At the same time, NP137 can significantly inhibit tumor growth and metastasis in some cancer patients, so netrin-1 can be used as a potential therapeutic target for the disease. However, although clinical and animal studies of the effects of netrin-1 and macrophage-derived netrin-1 on disease have been well defined, the specific downstream regulatory mechanisms of the associated receptors remain unclear. Therefore, the study of immune cell-derived netrin-1 remains a huge challenge to understand and overcome.

## Figures and Tables

**Table 1:** Macrophage-derived netrin-1 expression in inflammatory diseases.

Diseases	Sources	Expression	Receptors	Modes	Effects
Acute ischemic stroke	Brain tissue (microglia)	Increased	UNC5a	Autocrine	Promote microglia M2 polarization and reduce cell apoptosis, invasion, and migration
Acute lung injury (ALI)	Lung	Decreased	Neogenin (Neutrophils and monocytes)	Paracrine	Promote the recruitment of neutrophils, monocytes, and NK cells
Acute kidney injury (AKI)	Kidney	Decreased	Neogenin (Neutrophils and monocytes)	Paracrine	Promote the recruitment of neutrophils and monocytes
Diabetes	Diabetic wound	Increased	A2BR (Macrophages)	Paracrine	Promote angiogenesis and wound healing
Atherosclerosis	Artery (foam cells)	Increased	UNC5b (foam cells) and Neogenin (SMCs)	Autocrine and paracrine	Inhibit macrophages migration and induce SMC recruitment to the intima
Abdominal aortic aneurysms (AAA)	Abdominal aorta (macrophages)	Increased	Neogenin (vascular smooth muscle cells)	Paracrine	Promote transcription and calcium mobilization of matrix metalloproteinase 3 (MMP3)
Osteoarthritis	Bone tissue (osteoclasts)	Increased	DCC (nerve cells), UNC5b (osteoclasts), and A2BR	Autocrine and paracrine	Promote DRG neuron axon growth, subchondral bone sensory innervation, and osteoclast differentiation
Pulmonary Fibrosis	Lung tissue (macrophages)	Increased	DCC (nerve cells)	Paracrine	Remodeling of the adrenergic nerve and progression of fibrosis
Endometriosis	Uterine tissue (peritoneal macrophages)	Increased	CD146 (endothelial cells), Neogenin (nerve cells), DCC, and UNC5b (Schwann cells)	Paracrine	Promote angiogenesis and peripheral nerve regeneration and induce neuron regeneration

## Data Availability

Not applicable.
